# Bizarre Parosteal Osteochondromatous Proliferation (Nora’s Lesion) Affecting Carpal Bones of the Hand in a Middle-Aged Female: A Case Report

**DOI:** 10.7759/cureus.56772

**Published:** 2024-03-23

**Authors:** Vivek Tiwari, Samir Dwidmuthe, Suhas Aradhya Bhikshavarthi Math, Mainak Roy, Sachin R Chaudhari

**Affiliations:** 1 Department of Orthopedics, Apollo Sage Hospital, Bhopal, IND; 2 Department of Orthopedics, All India Institute of Medical Sciences, Nagpur, Nagpur, IND; 3 Department of Pathology, All India Institute of Medical Sciences, Nagpur, Nagpur, IND

**Keywords:** bone decortication, wrist swelling, hand swelling, nora's lesion, bizarre parosteal osteochondromatous proliferation

## Abstract

A 45-year-old woman complained of left wrist pain and swelling for two years accompanied by limited dorsiflexion. Plain X-rays revealed an abnormal bony mass in the carpal bones, further evaluated using computed tomography and magnetic resonance imaging. Upon confirmation of the benign nature surgical excisional biopsy of the lesion, the histopathology confirmed the diagnosis of Bizarre parosteal osteochondromatous proliferation (BPOP). The patient has remained pain-free and actively involved in her routine for the past two years. BPOP, affecting the carpal bones of the hand, are exceptionally rare occurrence. Attentive preoperative evaluation helps in diagnosis and to initiate measures to avoid recurrence.

## Introduction

Bizarre parosteal osteochondromatous proliferation (BPOP), also known as "Nora's lesion" described by Nora et al. in 1983, is relatively uncommon [1]. It typically manifests as an abnormal growth on the outer surface of a short bone in the hands or feet. BPOP is a non-cancerous condition and does not display any tendency for malignancy, but it does have a propensity to recurrence after surgical excision [2]. The proliferation of this lesion takes place on the outermost layer of the bone, although it does not have any connection to the normal part of the bone. Histopathological BPOP is characterized by an abnormal outgrowth from the outer bone surface, a combination consisting of bone, cartilage, and fibrous tissue [1,3,4].

BPOP progresses rapidly and has a high propensity for recurrence after surgical removal accounting for 50% [5]. Due to its aggressive characteristics and confusing presentation on histopathology, it is essential to differentiate it from malignant conditions such as chondrosarcoma, parosteal osteosarcoma, and conventional osteosarcoma, as well as benign conditions like florid reactive periostitis, myositis ossificans, periosteal chondroma, and osteochondroma [5]. Since its initial discovery, only a few cases documented in the literature, in this report, we present our experience with a single case of Nora's lesion.

## Case presentation

**Figure 1:**
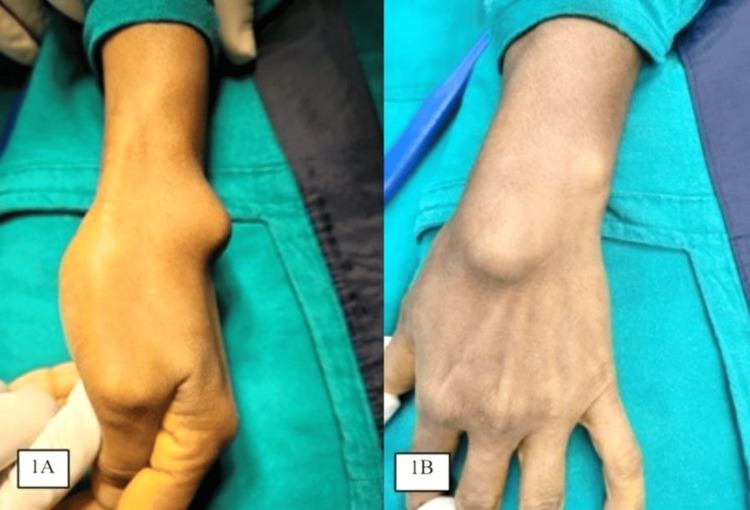
Pre-operative clinical image lateral view (A) and anteroposterior view (B) depicting swelling of 3x3 cm over the dorsum of the left hand

A 45-year-old woman came with a chief complaint of swelling on her left wrist over the dorsum that had been gradually progressive over the past two years with no history of antecedent trauma. Associated with pain, which aggravates on mobilization of the wrist and lifting heavy objects. On examination, the solitary mass was bony and hard in consistency, measuring about 3 cm by 3 cm (Figure 1), associated with tenderness. No skin adherence; wrist dorsiflexion is restricted terminally by twenty degrees due to a mechanical block of mass.

**Figure 2:**
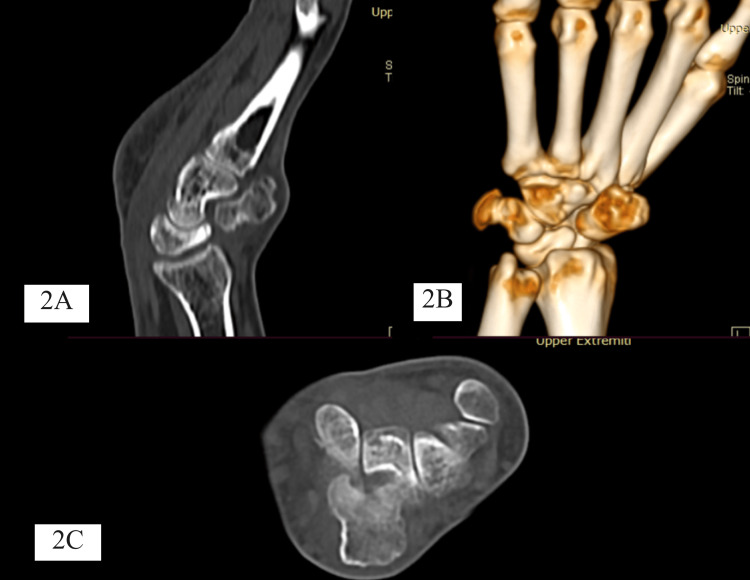
Pre-operative sagittal (A), 3D image (B), and axial cut sections (C) of computed tomography images depicting a left wrist bony lesion arising from lunate bone

Further evaluation using plain radiography and computed tomography revealed bony swelling arising from carpal bones over the dorsum aspect (Figure 2).

**Figure 3:**
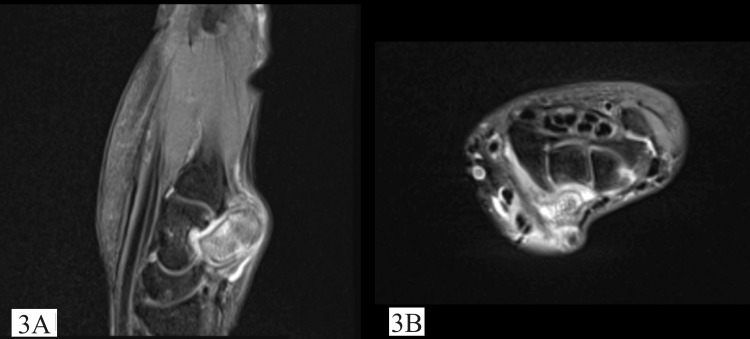
Pre-operative sagittal (A) and axial (B) magnetic resonance images depicting a left wrist bony lesion arising at the level of the lunate and triquetral proximally and capitate distally with increased signal intensity on the T2-weighted image (T2WI) image sequence

Magnetic resonance imaging scan showed a well-corticated mass approximately 1.5x1 cm sized lesion at the level of the lunate and triquetral proximally and capitate distally with hyperintense signal over proton density fat suppression and T2-weighted image sequence (Figure 3).

**Figure 4:**
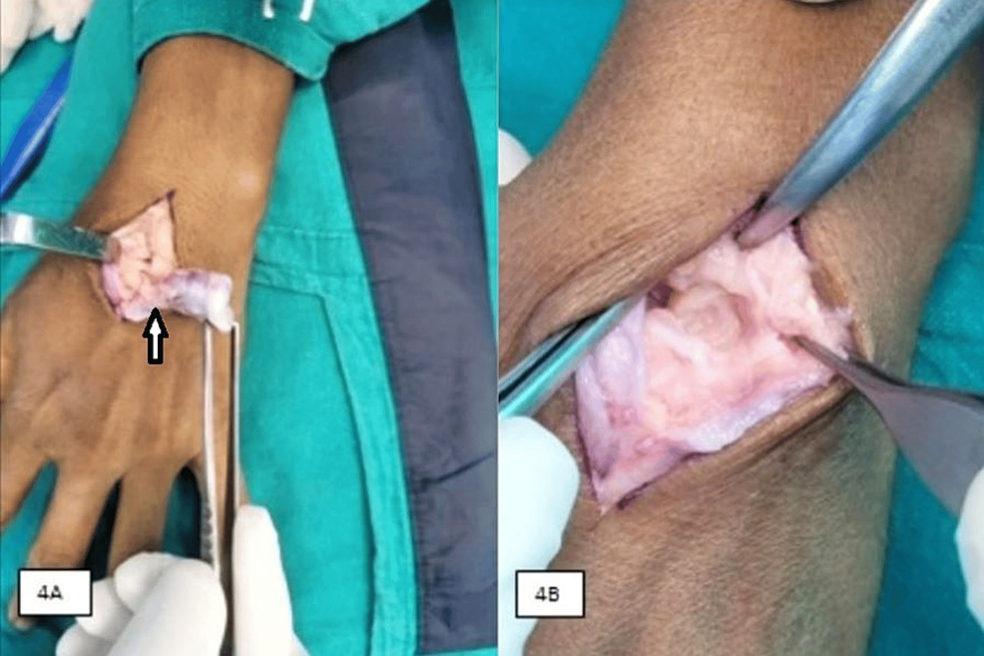
Intra-operative image depicting (A) removal of lobulated bony mass with stalk attached to lunate (arrowhead) and (B) bed of the lesion showing complete en-bloc excision of the mass

**Figure 5:**
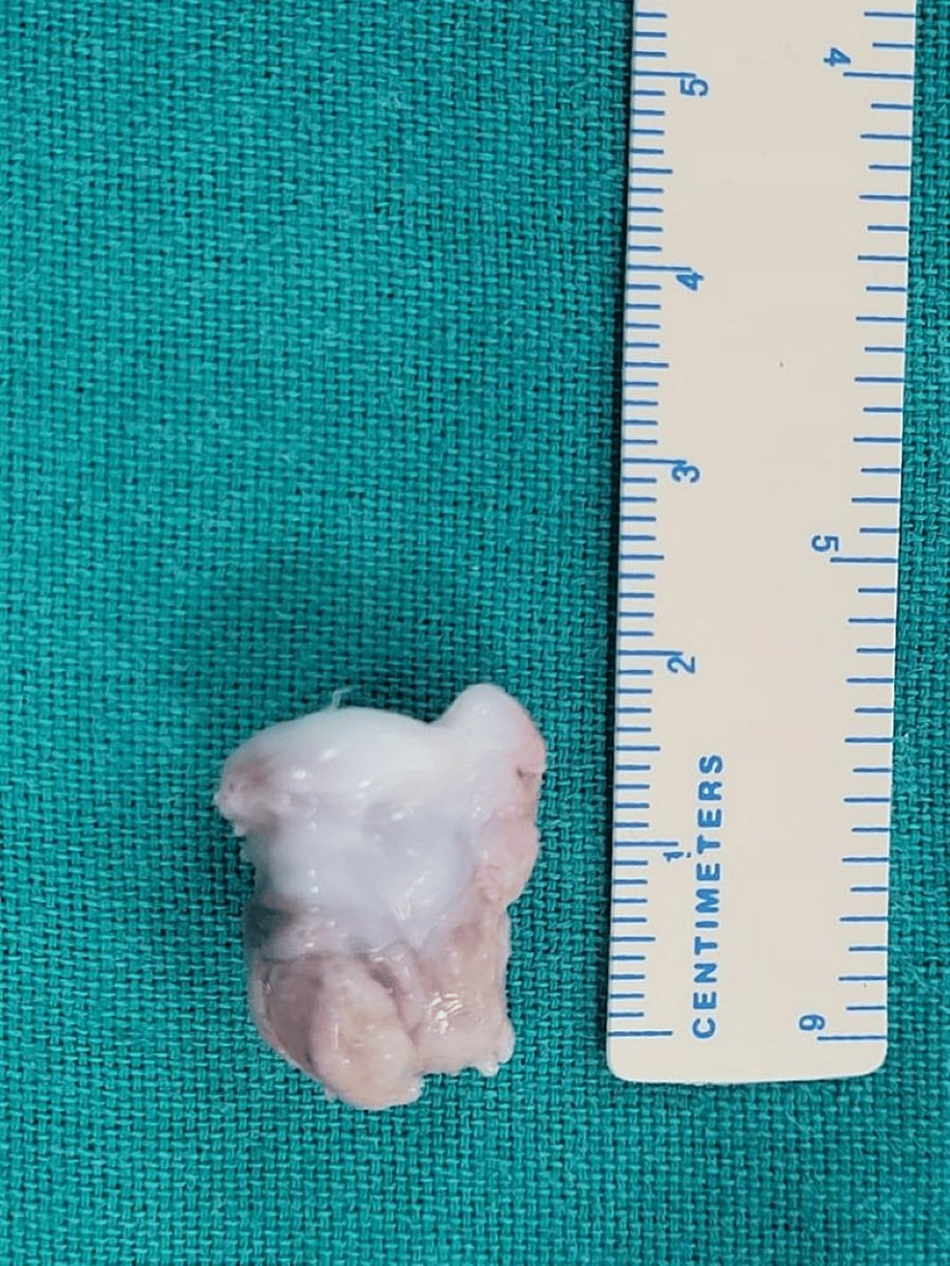
Post-operative excised lobular mass of size 18x10 mm

Probable differentials were ectopic calcification or a tumor; as the tumor laboratory profile was within normal limits, an excisional biopsy was planned. Dorsal wrist arthrotomy was performed using standard incision, the multi-lobular mass with pseudo-capsule excised along with periosteum (Figures 4, 5).

**Figure 6:**
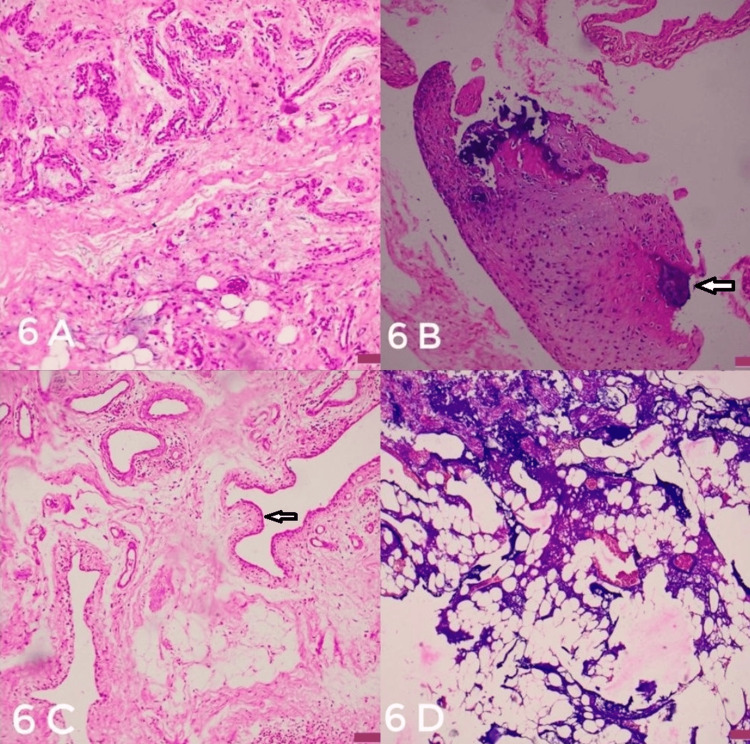
(A) Hematoxylin and eosin (H&E) staining of the tumor sections (magnification 10×), histopathology image showing thick-walled vessel with myxoid change (B) Hematoxylin and eosin (H&E) staining of the tumor sections (magnification 4×), cartilage with focal calcification (arrowhead), and enlarged nucleus, (C) Hematoxylin and eosin (H&E) staining of the tumor sections (magnification 10×), cuboidal epithelium of synovium (arrowhead), (D) Hematoxylin and eosin (H&E) staining of the tumor sections (magnification 4×), bony trabeculae with separated adipose tissue

**Figure 7:**
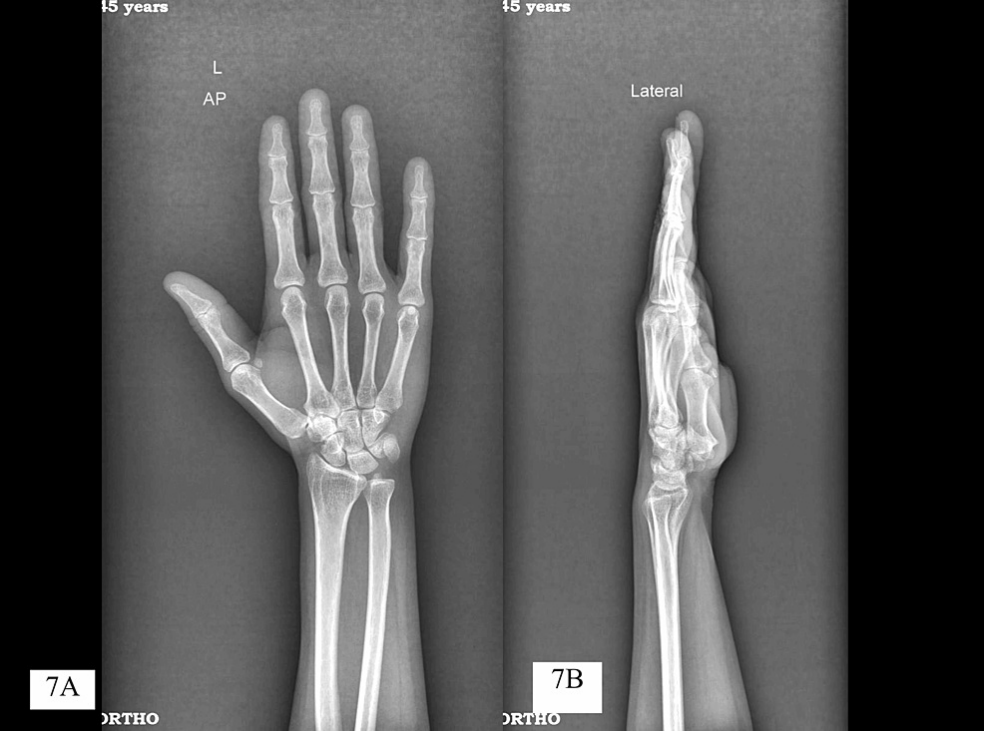
Two-year post-operative X-ray images (A) anteroposterior and (B) lateral view of hand showing no recurrence

**Figure 8:**
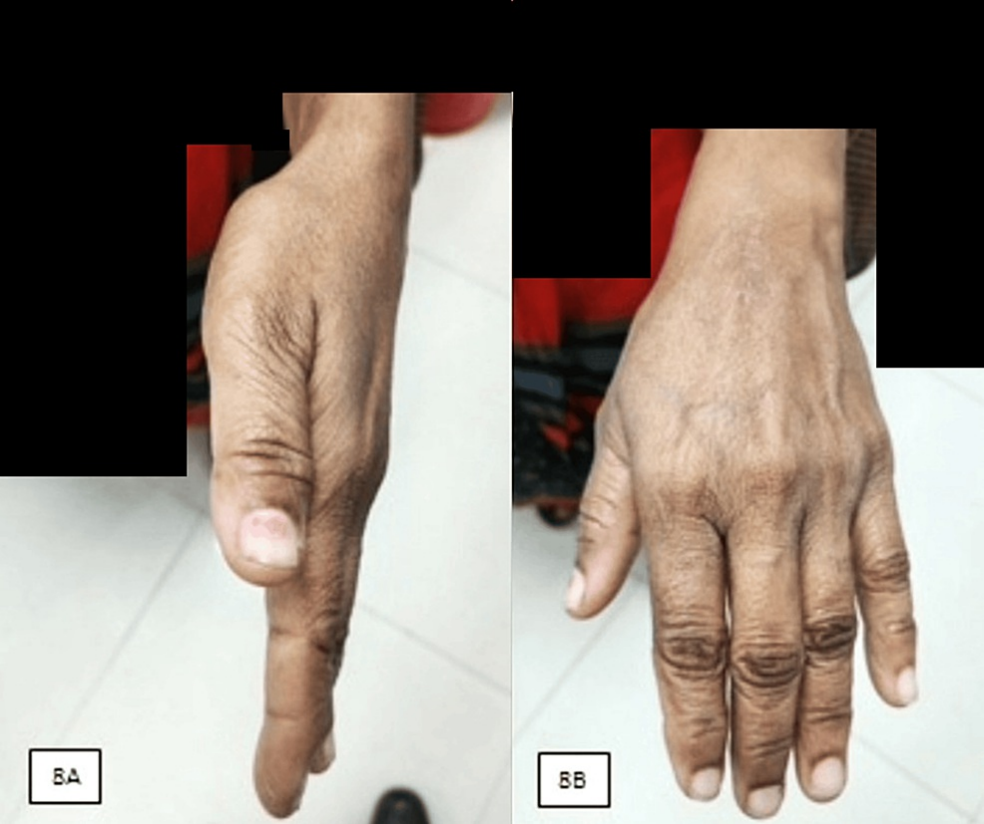
At two years of clinical follow-up, the lateral view (A) and anteroposterior view (B) of the hand show no signs of recurrence of swelling with a healthy scar

No adherence to surrounding bone or soft tissue structures was confirmed. Histopathological analysis (Figure 6) of the excised bony mass confirmed the presence of many irregular bony trabeculae separated by adipose tissue along with sparse collagenous tissue, surrounding soft tissue showed abundant fibro-collagenous tissue (lined by flat cuboidal epithelium suggestive of synovial tissue) along with adipose tissue and focal myxoid change with thick-walled blood vessel. Cartilaginous tissue with focal calcification and enlarged chondrocytes were noted. These histopathological findings were consistent with a diagnosis of BPOP. It has been two years after the surgery, and the patient is pain-free and has been able to carry out her daily routine (Figures 7, 8).

## Discussion

Bizarre parosteal osteochondromas of the bone, primarily occur on the proximal and middle phalanges, of metacarpal and metatarsal [6]. On rare occasions, they can affect long bones, often in the upper limbs, as well as the skull and jaw [7]. However, the presentation of carpal BPOP lesions is not described in the literature as far as our knowledge.

Nora's lesions typically occur in adults during their second and third decades of life, with an average age of 30 to 33 years with no gender discrimination [4]. Patients present with painful masses that gradually increase in size over several weeks or months. Although some patients may report joint stiffness or other mechanical symptoms. The exact cause of Nora's lesions remains unknown. Some theories correlate to the reparative process following trauma to the periosteum (the outer layer of bone), as observed in about 30% of cases in a study by Meneses et al [2]. However, our patient had no precedent history of trauma.

On plain X-rays, these lesions typically appear to arise from the bone's outer cortex but without any penetration [5]. They are often localized at the metaphysis with spiculated or irregular surfaces [5,8]. Computed tomography scans provide better bony morphology.

As a gross specimen, the lesion appears as a lump with a bumpy surface, covered in shiny cartilage and containing a hard inner core of bone [1]. Under a microscope, it displays unusual cartilage growth with a lot of cell activity, which gives a picture like Grade I or II chondrosarcoma [1]. There is also disorganized bone formation within the cartilage, along with irregularly calcified material and harmless bone cells, and there may be active growth of benign fibrous tissue [3,9]. Cancerous growths like chondrosarcoma, parosteal osteosarcoma, and conventional osteosarcoma, as well as non-cancerous conditions like reactive periostitis, myositis ossificans, periosteal chondroma, and osteochondroma, are possible differentials here. Therefore, it is crucial to approach with attention [3,5].

The recommended treatment is an en-bloc surgical excision along with scraping the surface of the underlying cortical bone to reduce the likelihood of recurrence [3,4]. However, there is a high chance of recurrence. Meneses et al. reported a 51% recurrence rate after the initial removal and a 22% recurrence rate after a second removal [2]. Most recurrences happened within two years of the index surgery [1,4,10].

## Conclusions

It is worth noting that BPOP involving the carpal bones is extremely rare. However, after surgical excision, there is a high chance of recurrence. A thorough evaluation is essential to isolate from its close differentials. Thorough excision followed by histopathological confirmation is vital. However, constant surveillance is crucial for recurrence.
